# Comprehensive evaluation of high dose methotrexate therapy: a retrospective observational trial

**DOI:** 10.1186/s43046-025-00324-9

**Published:** 2025-10-20

**Authors:** Hadeer Ehab Barakat, Kholood Ashraf El bahy, Sandy Victor Labib, Yasser Zakaria Aldesouky, Abdelrahman Ayman Ismail, Mohamed El Sayed Mohamed, Febrona louis sedky, Asmaa Mohamed abdelhady, Dalia Hamdy Gaballah, Doha Ashraf Ali, Passant Mohamed Refaat, Hasnaa Al Sayed Mohamed, Ahmed Zayed Mohamed, Salwa Selim Ibrahim, Ayman M Noreddin, Abdel-Moneim M. Osman, Esraa M. Abdelkeriem, Mohamed M Sayed-Ahmed, Riham M. Karkeet

**Affiliations:** 1 Ahram Canadian University, Giza, Egypt; 2 National cancer institute, cairo University, Giza, Egypt

**Keywords:** Methotrexate, Acute kidney injury, Hepatotoxicity, Length of hospital stay, Mortality

## Abstract

**Background:**

Methotrexate (MTX) is a commonly prescribed drug with both chemotherapeutic and immunosuppressive applications. However, when administered in high doses (HDMTX ≥ 500 mg/m^2^), it can lead to serious side effects, particularly nephrotoxicity and hepatotoxicity. Although 48-h MTX levels monitoring is fundamental for the evaluation of the risk of these toxicities, the relationship between MTX level and the actual clinical outcomes is not yet fully addressed. This study aims to evaluate the predictors of 48-h serum MTX levels and the toxicity profile associated with patients receiving HDMTX for management of cancer, with a particular focus on nephrotoxicity, hepatotoxicity, length of hospital stay (LOS), antimicrobial use, and 30-day mortality.

**Methods:**

A retrospective cohort study was conducted at the National Cancer Institute, Cairo University. Patients receiving HDMTX as part of their cancer treatment in the period from January 2022 to December 2024 were included. Data collection included patient demographics, administered MTX doses, 48-h serum MTX levels, medical and medication history, antimicrobials used, and recorded adverse effects. The outcome of the study encompassed the identification of predictors for 48-h MTX levels and their association with acute kidney injury (AKI), ICU admission, and LOS. In addition to the associations with hepatotoxicity, antimicrobial usage, and mortality. Statistical analysis was performed using SPSS version 26.0.

**Results:**

Among 143 patients, elevated 48-h MTX levels (≥ 1.28 μmol/L) were associated with pleural effusion (*P*-value 0.038), patients diagnosed with lymphoma (*P*-value 0.05), and increased antimicrobial use (*P*-value < 0.05). A significant association was found between HDMTX and the use of carbapenems, vancomycin and fluoroquinolones (*P*-value < 0.05). Non-significant relation was found between HDMTX and AKI as well as LOS. Hepatotoxicity was significantly more common in patients with osteosarcoma rather than hematological malignancies, while LOS was shorter in osteosarcoma cases compared to hematological malignancies.

**Conclusion:**

The serum levels of 48-h MTX are vital metrics of toxicity, as they determine the duration of hospitalization, the number of antimicrobials used, and the mortality rate. Thus, it is crucial to monitor these levels to reduce the complications associated with HDMTX usage.

## Introduction

Methotrexate (MTX) positions as an exceptional pharmacological tool with recognized achievement and effectiveness in treatment of several malignant tumors and immune diseases [1, 2]. Its anticancer influence is chiefly credited to the inhibition of dihydrofolate reductase, thus hampering the formation of activated folic acid [1]. Additionally, methotrexate-polyglutamate inhibits thymidylate synthase [1]. MTX has a favorable cost-effectiveness profile, but unfortunately highly toxic profile [2].

High-dose methotrexate (HDMTX) reflects a dose more than 500 mg/m^2^; this dose achieves significant efficacy and acceptable toxicity. HDMTX is used for central nervous system prophylaxis in patients with leukemia and high-risk lymphoma, as well as the treatment of leptomeningeal metastases, primary CNS lymphoma, and osteosarcoma. HDMTX is primarily eliminated through the kidneys, with up to 90% excreted unchanged in urine. HDMTX mandates two to three days of multiple leucovorin rescue doses to attenuate the toxic effect of MTX coupled with intense hydration and urinary alkalinization [3, 4]. Despite these supportive strategies, traditional estimates suggest that nephrotoxicity due to HDMTX occurs in 2% to 12% of all patients [5–7]. In addition, patients receiving MTX must be carefully monitored for side effects, including hepatotoxicity, neurotoxicity, myelosuppression, and mucositis [8, 9].

In order to monitor serum methotrexate levels throughout hospitalization and enable prompt interventions that may maximize clearance and minimize acute toxicity, HDMTX is typically administered in an inpatient setting. However, prolonged hospital stays are associated with dramatic raise in expenditure and increased risk of nosocomial infections. For patients, clinicians, and financiers, efforts to safely shorten length of stay (LOS) can potentially yield substantial benefits [8, 10]. Moreover, toxicities associated with HDMTX lead to greater morbidity, mortality, treatment interruptions and substandard clinical outcomes. Therefore, careful monitoring and quick intervention aiding methotrexate clearance attenuate toxicity, and allow subsequent HDMTX treatment.

Thus, the aim of this study was directed to predict the factors associated with high levels of 48 h MTX and comparatively assess the toxicity of HDMTX in different oncologic disorders and its effect on intensive care unit (ICU) admission, length of hospital stays, antimicrobials use, and 30 days mortality.

## Patients and methods

### Study design

A single center retrospective, observational cohort study was conducted with review of 143 patients’ files between January 2022 till December 2024; of patients receiving HDMTX for different oncological indications at the National Cancer Institute, Cairo University, Egypt. In a predefined table, patients’ files were used to extract all clinical data including patients' demographics (age, sex, body mass index, and body surface area), MTX dose and serum concentrations, medical history, current and past medications, laboratory results, length of hospital stay, toxicities, and mortality.

### High dose methotrexate treatment protocol

In the current study, the St. Jude TXV therapy protocol was used for patients with acute lymphoblastic leukemia (ALL). This included high-dose methotrexate (HDMTX) at 5 g/m^2^ for standard- and high-risk groups and 2.5 g/m^2^ for the low-risk group, administered as a 24-h infusion, along with triple intrathecal doses [11]. For non-Hodgkin lymphoma (NHL), treatment followed the FAB-LMB 96 protocol, which stratifies patients into three risk groups (A, B, and C) according to disease stage, bone marrow involvement, and central nervous system involvement. HDMTX was administered over 4 h (3 g/m^2^ for group B and 8 g/m^2^ for group C), combined with methotrexate and triple intrathecal therapy [12]. Osteosarcoma patients were treated according to the EURAMOS protocol, which includes HDMTX at 12 g/m^2^ over a 4-h infusion. Across all patient groups, growth factors were not used to aid recovery from myelosuppression [13].

### Eligibility criteria

The current study included all patients receiving HDMTX (defined as ≥ 500 mg/m2) between 2022 and 2024 for cancer treatment. Patients were excluded from the study if they received multiple HDMTX doses during a single hospitalization or if their methotrexate level 48 h after administration was not recorded.

### Study outcomes

The primary outcomes of this study were to determine the predictors of 48-h MTX level at the cutoff point of 1.28 μmol/L[14]. In addition, the relation between the 48-h MTX level with AKI, length of hospital stays, and ICU admission. The secondary outcomes were to determine the association of HDMTX with other toxicities such as hepatotoxicity and myelosuppression. In addition, the association with medications used during hospitalization, disease progression, and 30-day mortality were also reported. All toxicities were defined according to Common Terminology Criteria for Adverse Events (CTCAE) Version 5.0.

### Ethical consideration

The study was approved by the Institutional Review Board (IRB) of the National Cancer Institute, Cairo University (CB2502-002–003-197). The study was conducted in accordance with the Declaration of Helsinki, Good Clinical Practice norms, as well as local and national regulatory requirements.

### Statistical analysis

Data was analyzed using IBM SPSS advanced statistics (Statistical Package for Social Sciences), version 26 (SPSS Inc., Chicago, IL). Numerical data were described as mean and standard deviation. Categorical variables were presented as numbers and percentages. To examine the relationship between 48-h MTX levels and AKI, hepatotoxicity, Length of hospital stay, ICU admission, and 30 days mortality, chi-squared tests were used for categorical variables and the Kruskal–Wallis test was employed for nonparametric data. Logistic regression was conducted to predict high levels 48-h MTX and its complications of AKI, hospital stay, ICU admission, and mortality. In addition logistic regression is used to predict toxicities and complications according to the type of tumor. Chi-squared test was used to assess the relation between antimicrobials administered and the associated toxicities. *P*-value ≤ 0.05 was considered significant.

## Results

### Demographic data and patients’ characteristics

As shown in Table 1, a total of 143 patients were observed who received high dose methotrexate in their first cycle in the period between 2022 and 2024. 73% of participants were aged less than 18 years, 67% were males and 62% were diagnosed with osteosarcoma. The mean body surface area was 1.36m^2^ (1.28–1.43), the mean dose of methotrexate was 11.42 gm/m^2^ (10.24–12.61), and the mean of hospital stay was 9.8 days (8.47–11.19). Only one patient suffered cardiac comorbidity, three patients suffered hypertension and one patient had diabetes mellitus.

**Table 1:** Demographic data and patients’ characteristics

Variable	N (%)
Age	
< 18 years	105 (73%)
> 18 years	38 (27%)
Sex	
Male	96 (67%)
Female	47 (33%)
BMI (Kg/m^2^)	
Underweight	67 (47%)
Normal	52 (37%)
Overweight	12 (8%)
Obese	12 (8%)
Diagnosis	
Osteosarcoma	89 (62%)
Acute Lymphoblastic Leukemia	33 (23%)
Non-Hodgkin's Lymphoma	21 (15%)
48 h- Methotrexate level	
< 1.28 μmol/L	122 (85.3%)
> 1.28 μmol/L	21 (14.7%)

### Predictors of high dose methotrexate

Univariate analysis showed that age, sex, body surface area, baseline serum creatinine, and dose of methotrexate have no significant effects on 48-h MTX level. The analysis revealed a significant increase in pleural effusion with 48-h MTX ≥ 1.28 μmol/L (OR 13.6; *P*-value 0.038) as shown in Table 2. In addition, the diagnosis of patients with lymphoma is a significant predictor of 48-h MTX ≥ 1.28 μmol/L (OR 3.16; *P*-value 0.05).

**Table 2:** Univariate analysis of the predictors of 48 h MTX level

Variable	Odds Ratio	95% CI		*P*-value
Age	1.187	(0.403	3.496)	0.756
Sex	1.025	(0.384	2.739)	0.701
Body surface area	1.098	(0.351	3.430)	0.872
Baseline serum creatinine	1.177	(0.477	2.909)	0.723
Dose of methotrexate	1.050	(0.890	1.019)	0.154
Pleural effusion	13.6*	(1.161	159.32)	0.038
Diagnosis				
Leukemia	1.411	0.444	4.486	0.056
Lymphoma	3.16*	0.998	10.009	0.05
Osteosarcoma	1			

^*^P-value < 0.05, significant

Analysis of the medication history of the patients including non-steroidal anti-inflammatory drugs (NSAIDs), proton pump inhibitors (PPIs), antimicrobials, and antigout medications showed that both proton pump inhibitors and antigout medications increased significantly the 48-h methotrexate level (*P*-value 0.01).

### Relation between level of methotrexate and toxicity

Non-significant increasing association was detected between 48-h MTX ≥ 1.28 and acute kidney injury (OR 2.4; *P*-value 0.092) as shown in Table 3.

**Table 3:** Univariate analysis of methotrexate level and its complications

Toxicity	MTX < 1.28 μmol/L	MTX > 1.28 μmol/L	Odds Ratio	95% CI		*P*-value
Acute kidney injury	21 (17.2%)	7 (31.8%)	2.4	(0.150	1.155)	0.092
Hepatotoxicity	41 (37.9%)	9 (42%)	1.47	(0.255	1.813)	0.441
ICU admission	2 (1.6%)	1 (4.8%)	3.01	(0.260	34.650)	0.379
Progression	17 (16.7%)	2 (11.1%)	0.63	(0.131	2.973)	0.555
30 days mortality	2 (1.6%)	1 (4.8%)	3.03	(0.029	3.850)	0.379

### Length of hospital stay

**Fig. 1:**
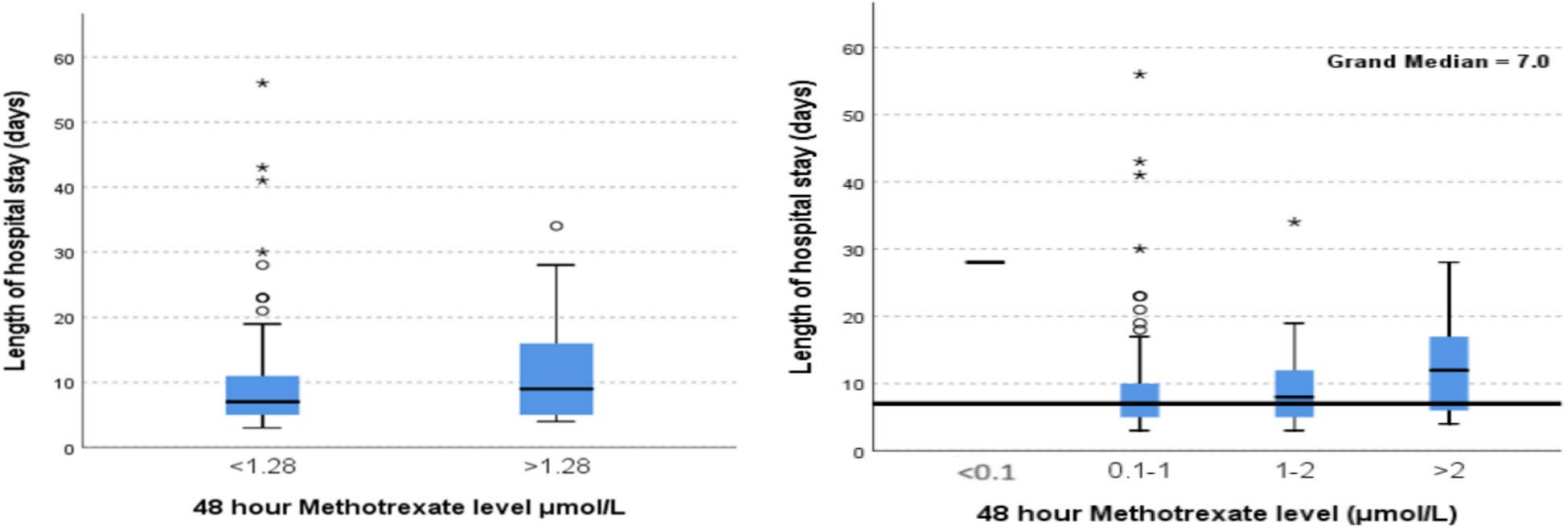
Relation between methotrexate level and median length of hospital stay in days

The median hospital stay was slightly longer in patients with methotrexate levels ≥ 1.28 (9 days) compared to those with levels < 1.28 (7 days), although this difference was not statistically significant (*P*-value 0.118), as shown in Fig. 1. In addition, the median length of hospital stay increases from 7 days at 48-h MTX level of 0.1–1 μmol/L to 8 days at MTX level of 1–2 μmol/L to 13.5 days at MTX level of > 2 μmol/L.

### Toxicities and complications associated with hematological malignancies versus solid tumor

**Fig. 2:**
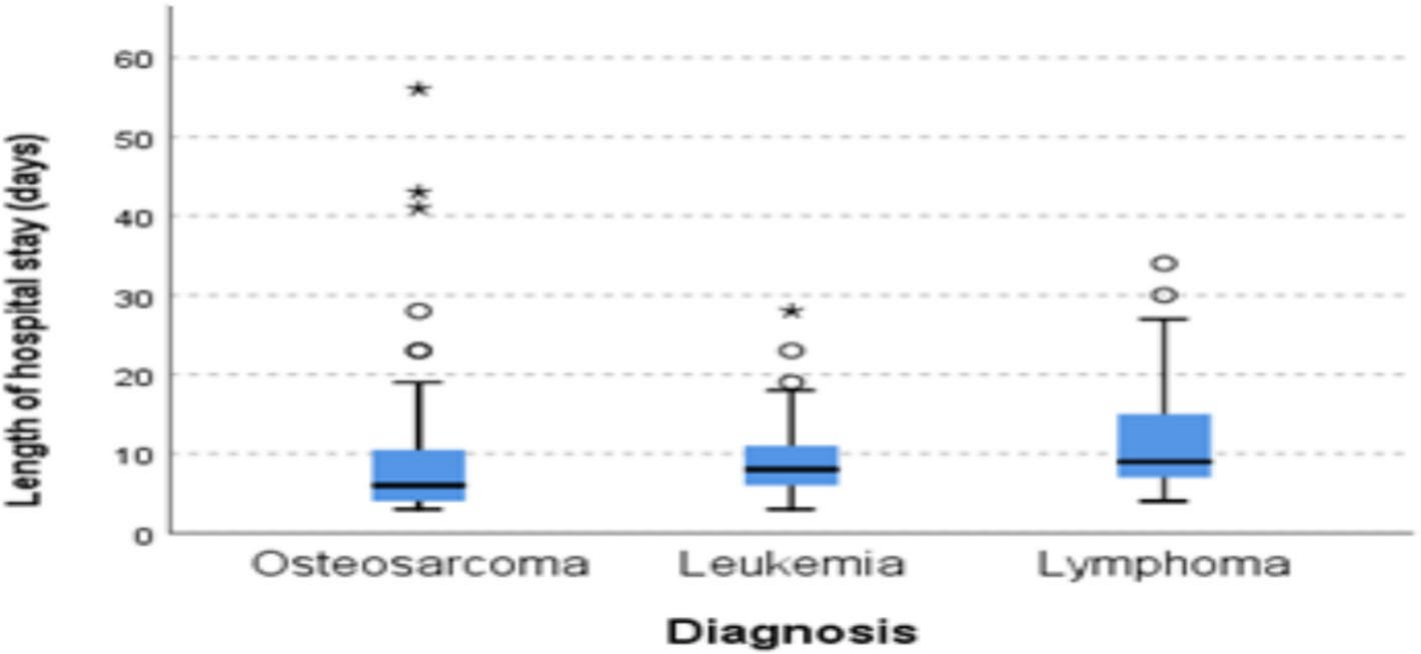
Relation between the type of tumor and median length of hospital stay in days

As shown in Table 4, toxicity outcomes varied between solid tumors and hematological malignancies. Hepatotoxicity was significantly more common in solid tumors (48.3%) than in hematological malignancies (12.9%) (OR 3.254; 95% CI: 1.071–9.891; *P*-value 0.038). Other outcomes including acute kidney injury, disease progression, and 30-day mortality, showed no statistically significant differences between the two groups. Figure 2 shows that patients with osteosarcoma had a shorter median hospital stay compared to those with leukemia and lymphoma, with lymphoma showing the greatest variability in duration of stay.

**Table 4:** Univariate analysis of the association between the tumor type and its complications

Toxicity	Solid tumors (Osteosarcoma)	Hematological malignancies	Odds Ratio	95% CI		*P*-value
Acute kidney injury	15 (16.9%)	13 (24.02%)	0.649	(0.206	2.043)	0.460
Hepatotoxicity	43 (48.3%)	7 (12.9%)	3.254*	(1.071	9.891)	0.038
Progression	18 (20.22%)	1 (1.8%)	5.684	(0.709	45.61)	0.102
30 days mortality	1 (1.12%)	2 (3.7%)	9.263	(0.798	107.466)	0.075

^*^P-value < 0.05, significant

### Medications used during hospital stay

**Fig. 3:**
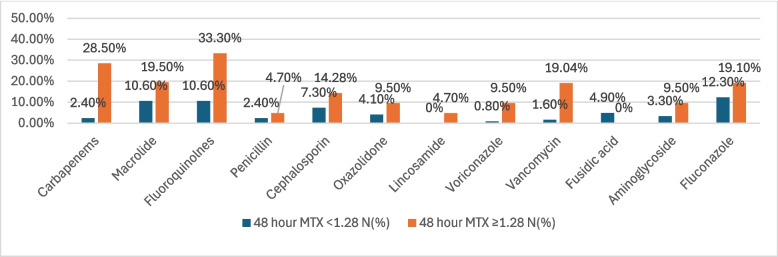
The antimicrobial use in relation with 48-h MTX dose

Figure 3 shows that most antimicrobials used have increased in association with 48 h MTX level ≥ 1.28 μmol/L. The use of carbapenems, vancomycin and fluoroquinolones increased significantly with MTX level ≥ 1.28 μmol/L during the hospital stay (*P*-value < 0.05).

No statistically significant association was observed between antimicrobial use and nephrotoxicity or hepatotoxicity as shown in Table 5.

**Table 5:** The association between toxicities and the use of non-chemotherapeutic medications

Medication	Number of patients receiving Medications	Patients with Nephrotoxicity N (%)	*P*-value	Patients with Hepatotoxicity N (%)	*P*-value
Carbapenems	9	3 (33.3%)	0.283	1 (11.11%)	0.072
Macrolide	17	2 (11.7%)	0.387	4 (23.5%)	0.208
Fluoroquinolones	20	5 (25.0%)	0.510	7 (35.0%)	0.259
Penicillin	4	1 (25.0%)	0.782	1 (25.0%)	0.829
Cephalosporin	12	2 (16.6%)	0.790	4 (33.3%)	0.831
Oxazolidone	7	2 (28.5%)	0.539	1 (14.2%)	0.162
Lincosamide	1	0 (0.0%)	0.620	0 (0.0%)	0.419
Voriconazole	3	0 (0.0%)	0.388	1 (33.3%)	0.829
Vancomycin	6	1 (16.6%)	0.854	2 (33.3%)	0.757
Aminoglycoside	4	2 (50.0%)	0.386	3 (75.0%)	0.585
Fluconazole	19	4 (21.1%)	0.862	4 (21.1%)	0.208
Proton pump inhibitor	20	2 (10.0%)	0.224	4 (20.0%)	0.381
NSAID	6	1 (16.67%)	0.854	2 (33.3%)	0.997
Antigout	14	6 (42.8%)	0.082	4 (28.5%)	0.381

^*^P-value < 0.05, significant

## Discussion

In the current study, patients received high dose methotrexate from 2022 till 2024 revealed a significant association between the patients with pleural effusions and elevated 48-h MTX levels based on the cutoff value of ≥ 1.28 μmol/L [14]. In agreement with previous studies, patients diagnosed with lymphoma experienced significantly higher 48 h MTX levels [5–7] Unlike Donoghue et al., age, sex, and BSA were not found as predictors of high methotrexate level [14].

Reviewing the medical and medication history of the patients showed that both proton pump inhibitors (PPI) and antigout medications have significantly increased MTX level. Evidence indicates that the concurrent use of methotrexate, particularly at high doses, with PPIs and antigout medications may reduce methotrexate clearance. This reduction can result in increased serum levels of methotrexate and/or its metabolite hydroxymethotrexate, potentially causing methotrexate-related toxicities [15, 16].

The overall incidence of AKI in this study was 19.5%. Although it is notably higher than the traditionally reported rates of 2% to 12% [5], it is similar to that observed by Donoghue et al., in patients with lymphoma receiving a high dose of methotrexate [14]. In contrast, Wiczer et al. documented a significantly higher nephrotoxicity rate of 39% in their retrospective study of high dose MTX [17]. Additionally, only numerical increase in acute kidney injury and hepatotoxicity was observed with MTX level ≥ 1.28 μmol/L, the lack of significance could be attributed to the small sample size. Furthermore, the median hospital stay length increased with higher MTX levels, are consistent with the findings of Donoghue et al. [13]. Specifically, the median hospital stay was non-significantly longer at 9 days for methotrexate levels ≥ 1.28 compared to 7 days for levels < 1.28. Moreover, the median hospital stay extends from 7 days for a 48-h MTX level of 0.1–1 μmol/L to 8 days for an MTX level of 1–2 μmol/L and further increases to 13.5 days for a MTX level exceeding 2 μmol/L. These findings carry significant short-term implications for hospitalization and ICU admission, potentially delaying subsequent MTX cycles and increasing healthcare costs.

In the current study, both toxicity profiles and hospital stay durations varied according to tumor type. Hepatotoxicity was significantly more common in patients with solid tumors compared to those with hematological malignancies which might be due to the fact that treatment regimens for solid tumors carry a higher risk of liver-related adverse effects. Langman et al., concluded that the cases of methotrexate toxicity might actually be due to nonalcoholic steatohepatitis [18]. Another possible explanation for this finding aligns with the study by Abe K. et al., which identified female sex as a risk factor for hepatotoxicity in patients with osteosarcoma who received MTX [19]. In the currently studied cohort, 38% of osteosarcoma patients were female compared to only 24% in the hematological malignancies group, potentially contributing to the higher rate of hepatotoxicity observed in the solid tumor group. The influence of female sex hormones, along with sex-related differences in methotrexate pharmacokinetics, may partially account for this disparity. In agreement with Wang et al. the co-administration of PPI and NSAIDs have limited non- significant effects on acute hepatotoxicity [20]. On the other hand, other toxicities such as acute kidney injury, disease progression, and 30-day mortality were more frequent in one group or another, but these differences did not reach statistical significance.

Regarding the length of hospital stay and in agreement with previous studies [21, 22], patients with osteosarcoma had a shorter median length of stay compared to those with leukemia and lymphoma. Lymphoma patients showed the greatest variability in hospitalization duration, mostly due to combination of HDMTX with other myelosuppressive chemotherapeutic agents resulting in longer hospital stay.

In this context, the univariate analysis showed significant increase in some antimicrobials with MTX levels ≥ 1.28 μmol/L for the management of nosocomial infections [23, 24]. Notably, the use of carbapenems, vancomycin, and fluoroquinolones showed a significant increase during hospitalization at MTX levels ≥ 1.28 μmol/L (*P*-value 0.05). Carbapenems are considered the antibiotics of last resort in many regions among β-lactams for human treatment due to their safety and effectiveness in addressing multidrug-resistant (MDR) gram-negative bacterial infections. However, over the past decade, the increasing prevalence of carbapenem resistance has presented a significant challenge for clinicians in managing both community- and healthcare-associated infections [25]. Comprehensive and targeted antimicrobial resistance (AMR) surveillance, with a focus on carbapenem resistance, is urgently needed to create and implement effective national policies aimed at preserving the effectiveness of carbapenems as last-resort antibiotics [25].

Methotrexate is primarily eliminated by the kidneys and in a lesser extent by the liver. Thus, impaired elimination, which can lead to serious toxic effects, may occur due to renal dysfunction caused by inadequate alkaline hydration, consumption of acidic beverages, or drug-drug interactions [26, 27]. The co-administration of some antimicrobials has been shown to delay methotrexate elimination via the kidneys, potentially resulting in severe toxicity. These include the penicillins piperacillin, alone or combined with tazobactam, oxacillin, amoxicillin, mezlocillin, the fluoroquinolone ciprofloxacin, the glycopeptide vancomycin and the streptogramin pristinamycin [26]. Moreover, delayed methotrexate clearance may also be attributed to renal dysfunction secondary to sepsis. In the current study, most antimicrobials were not significantly associated with nephrotoxicity or hepatotoxicity.

The key strengths of this study is its comprehensive evaluation of multiple clinically relevant outcomes associated with HDMTX therapy, including 48-h serum methotrexate levels, nephrotoxicity, hepatotoxicity, antimicrobial use, and hospital stay duration. The inclusion of a relatively diverse patient cohort across different cancer types enhances the generalizability of the findings. Furthermore, the study provides a detailed analysis of the relationship between methotrexate levels and concomitant medication use, particularly antimicrobials, offering valuable insights into potential drug interactions. By incorporating both biochemical data and clinical outcomes, the study delivers a more integrated understanding of HDMTX toxicity in real-world settings, which can support more individualized and safer patient care strategies. However, the limitations of the current study include its retrospective nature and the missed data of some patients.

## Conclusion

This study focused on the role of 48-h serum MTX levels as a predictive marker for toxicity in patients undergoing HDMTX. Elevated MTX levels at 48 h were associated with significant increase in antimicrobials and numerical increase in the incidence of nephrotoxicity and length of hospital stays. These findings showed the crucial importance of monitoring serum MTX levels particularly for identifying patients at higher risk of toxicity and complications. Further research is needed to explore the optimal thresholds for serum MTX levels, as well as to establish more precise protocols for mitigating the associated risks of HDMTX therapy. Ultimately, this study supports the need for a tailored treatment approaches balancing the efficacy of HDMTX with patient safety considerations to minimize potential life-threatening complications.

## Data Availability

The data that support the findings of this study are available on request from the corresponding author.
